# Social stress and depression during pregnancy and in the postnatal period in British Pakistani mothers: A cohort study

**DOI:** 10.1016/j.jad.2012.02.009

**Published:** 2012-11

**Authors:** Nusrat Husain, Kennedy Cruickshank, Meher Husain, Sarah Khan, Barbara Tomenson, Atif Rahman

**Affiliations:** a School of Community-based Medicine, University of Manchester, Manchester, UK; b Culture and International Mental Health Research Group, Lancashire Care NHS Foundation Trust, Lancashire, UK; c St Thomas' Hospital, King's College, London, UK; d University of Manchester, Manchester, UK; e Central Manchester Foundation Trust, Manchester, UK; f Institute of Psychology, Health and Society, University of Liverpool, Liverpool, UK

**Keywords:** Social stress, Pregnancy, Postnatal, Ethnic minority, EPDS

## Abstract

**Background:**

Depressive disorders are common and disabling among perinatal women. The rates are high in ethnic minority groups. The causes are not known in British Pakistani women. The aim of this study was to estimate the rates, correlates and maintaining factors of perinatal depression in a Pakistani sample in UK. The design used was a cross-sectional two phase population based survey with a prospective cohort study.

**Methods:**

All women in 3rd trimester attending antenatal clinic were screened with the Edinburgh postnatal depression scale (EPDS). Women scoring 12 or more on EPDS and a random sample of low scorers were interviewed using the Schedules for Assessment in Neuropsychiatry (SCAN) and the Life Events and Difficulties schedule (LEDS). Social support was assessed with the Multidimensional Scale for Perceived Social Support (MSPSS). They were reassessed 6 months after the delivery using the same measures.

**Results:**

The weighted prevalence of depression was 16.8%. Depressed mothers had more marked non health difficulties (housing, financial and marital). They had less social support and were socially isolated. Marked social isolation and marked non-health related difficulties were independent predictors of depression. Analyses of all the possible risk factors, comparing 26 persistent depressed with 27 depression resolved group showed significant differences in the MSPSS subscales between the two groups.

**Limitations:**

The study lacked inter-rater reliability testing between the individuals carrying out diagnostic interviews. The study sample did not accurately represent the general population and information about the origins of depression in this group of mothers was limited.

**Conclusion:**

Depression in British Pakistani mothers is associated with social isolation, poor social support and severe and persistent social difficulties. The findings will have implications in planning suitable services for this group.

## 1 Introduction

Depression is a common and disabling psychiatric disorder in the UK and worldwide (Alonso et al., 2004; Demyttenaere et al., 2004; Kessler et al., 2005; Weich and Araya, 2004). It is 1.5 to 3 times more prevalent in women than men, and is particularly common among women of child-bearing age. In the UK the economic burden of depression was estimated to be £9 billion in 2000 (Thomas and Morris, 2003). In a recent population-based project (Gater et al., 2009), the depression prevalence was higher in women of Pakistani family origin living in the UK (31.0%) than in white European women (12.9%), confirming our previous findings (Husain et al., 1997).

Postpartum depression affects approximately 10–15% mothers in Western societies (O'Hara and Swain, 1996) and up to 25% in Pakistan (Rahman et al., 2004). Maternal depression is a treatable disorder, which negatively influences the psychological and cognitive development of children (Cooper and Murray, 1998). There is growing evidence from developing countries that it has negative effects on growth and physical health (Patel et al., 2003; Rahman et al., 2004). Chronic depression carried a greater risk for poor outcome than episodic depression. Based on current research, the strongest predictors of postpartum depression are experience of depression or anxiety during pregnancy, a previous history of depressive illness, recent experience of a stressful life event and those who perceive they have low social support (Robertson et al., 2004).

Some other studies investigating post-partum depression in ethnic minorities have also reported similar results. High scores on the Edinburgh Postnatal Depression Scale (EPDS) have been associated with 2 independent factors, namely the participant being non-White (especially Asian) and also being born in a non-English speaking country (Onozawa et al., 2003). Qualitative interviews with Bangladeshi women living close to London have revealed that the women felt rejected and isolated after giving birth, receiving very little social support from their husbands or the extended family (Parvin et al., 2004). The participants were able to distinguish between emotional and physical distress and felt that one could influence the other. The women were reluctant to talk to the medical practitioners about their mental health as they believed that the GPs were only concerned with their physical symptoms (Parvin et al., 2004).

Templeton et al. (2003) also examined the experiences of women from Black and ethnic minority communities in relation to postnatal depression. Some women showed a lack of understanding about the illness, whereas even though others knew of depression they were unable to identify their own symptoms. Some women talked about repeated and untreated episodes of postnatal depression, with the language barrier being a major contributor to the lack of medical support. Women said they were unaware of the support being provided by primary care services while some deemed the care being provided as inadequate, in terms of the early discharge after giving birth (Templeton et al., 2003). Recently Almond and Lathlean (2011) have reported that there is inequity in the provision and access to postnatal depression services. Their results indicate that women in ethnic minority groups are not always assessed for post-partum depression by the health visitors and do not receive the same support as the majority white women.

The experiences of women of British Pakistani women need to be considered, as their interactions with, and access to, health care services, experience of social adversity, and opportunities for social networks and support may differ significantly from other groups. Lack of social support is a well-established risk factor for postpartum depression, and Pakistani women in the UK may be at higher risk of depression because they are culturally and physically separated from their support systems.

### 1.1 Aims

The aims of this study are to estimate rate of perinatal depression in British Pakistani women and to study the risk factors associated with onset and persistence of depression.

This is part of a larger MRC funded study which prospectively looked at the growth, physical morbidity and psychological development of a representative sample of the infants of mothers of Pakistani origin who either had depression, or who were psychologically healthy.

## 2 Methods

### 2.1 Design

This was a longitudinal study with a two phase (Dunn, 2000) assessment procedure (antenatal) followed by a six month (postnatal) prospective cohort of depressed and non depressed mothers.

### 2.2 Recruitment of subjects

The sample of women included British Pakistani pregnant women attending antenatal clinics at two different hospitals in the North West of England.

### 2.3 Antenatal assessments: phase-1

All women attending the antenatal clinic were approached by a bilingual research assistant and invited to take part in the study; an information leaflet (both in English and Urdu) was given to explain the study. Verbal information was given to those who were unable to read. All the women who were in their third trimester of pregnancy and gave informed consent were screened at the antenatal clinic with the Edinburgh Postnatal Depression Scale (EPDS) (Cox et al., 1987), for depression (phase 1 interview). An Urdu translated version of the EPDS (Rahman et al., 2004) was completed by women who were unable to read English, and for those who were illiterate the research assistant who was fluent in Urdu would read the EPDS word for word for the participant and they would respond accordingly. Women with multiple pregnancies, or diagnosed physical or learning disability were excluded.

### 2.4 Antenatal assessments: phase-2

Women scoring 12 or more on the EPDS and a random sample of low scoring women were invited to attend phase-2 interviews, which included a diagnostic interview using the Schedules for Clinical Assessment in Neuropsychiatry (SCAN) (WHO, 1994) and the Life Events and Difficulties schedule (LEDS) (Brown and Harris, 1978) to measure social stress. These Instruments are available in Urdu and have been used in Pakistan (Husain et al., 2000) and with people of Pakistani origin in the UK (Gater et al., 2009).

Demographic details including some acculturation factors like 1st generation, 2nd generation, and proficiency in English etc. were also obtained. Social support was measured using *Multidimensional Scale of Perceived Social Support* (MSPSS) (Akhter et al., 2010; Husain et al., 2006; Zimet et al., 1988). All phase-2 interviews were conducted within 4 weeks of screening, and most were conducted at home except for a few women who preferred to be interviewed at the clinic. All interviews were conducted in the preferred language of the individual. All the researchers were provided with rigorous training in both English and Urdu for EPDS (Cox et al., 1987), LEDS (Brown and Harris, 1978) and SCAN (WHO, 1994). Steps were taken to avoid potential bias; participants were not interviewed by the researcher who had initially screened them in the first phase of the study.

### 2.5 Follow up assessments

All women who completed both phases of the antenatal assessments were invited for a follow up interview when their baby was 6 months old, during which the SCAN and LEDS interviews were repeated. Women with post-partum or other psychosis, and infants born prematurely, with congenital deformity, physical or mental handicap were excluded.

### 2.6 Psychiatric instruments

#### 2.6.1 Edinburgh Postnatal Depression Scale (EPDS) (Cox et al., 1987)

EPDS is a screening tool to detect postnatal depression. This is a 10 item self-report questionnaire with four possible responses. The response categories are scored 0, 1, 2, 3 according to increased severity of symptoms, items 3, 5–10 are reverse scored (i.e. 3, 2, 1, and 0). The total score is calculated by adding together the scores for each of the 10 items. We have previously used EPDS in Pakistan with good psychometric properties (Husain et al., 2006; Rahman et al., 2003). A score of ≥ 12, the most commonly used cut-off, was used to distinguish cases from non-cases.

#### 2.6.2 Schedule for Clinical Assessment in Neuropsychiatry (SCAN)

SCAN is a set of instruments aimed at assessing, measuring and classifying the psychopathology and behaviour associated with the major psychiatric disorders of adult life (WHO, 1994). The SCAN is administered by trained interviewer who can record the responses either directly into the computer or on paper. The symptoms should have been experienced by the respondent in a specified period (four weeks for this study). The symptoms are rated according to the severity and entered into the computer programme. The data is processed to give a diagnosis based on ICD-10 and DSM-IV. The SCAN has been used in Pakistani population in the UK (Gater et al., 2009) and in Pakistan (Rahman et al., 2003).

#### 2.6.3 Life Events and Difficulties Schedule (LEDS)

This is an instrument for the assessment of social adversity and isolation. It covers domains of family, work, health, and relationships, to establish any life events and difficulties that may have occurred or be present, in the twelve months prior to the date of interview. Each question has several probes to gain a full understanding of what has happened, date when it occurred who was involved and to what extent. Probing in this way is continued until it is felt that a complete and clear picture of the event or difficulty is gained. The interview also covered a brief assessment of education, literacy, fluency in English, religious practise and an objective measure of social adversity, such as income (Brown and Harris, 1978). There are two LEDS manuals, one for rating events and the other for rating difficulties. The guidelines are given to decide whether an event or difficulty is to be included.

The LEDS has been used in Pakistani population in UK (Gater et al., 2009; Husain et al., 1997) and in Pakistan (Husain et al., 2000). There are certain limitations of the LEDS manual such as many of its examples of events and difficulties are rooted in the 1970s Camberwell population which was mainly White Europeans living in the UK. We have developed an appendix to the manual which has examples from the previous studies with people of Pakistani origin.

#### 2.6.4 Multidimensional Scale of Perceived Social Support (MSPSS) (Zimet et al., 1988)

This 12-item scale measures the subjective assessment of social support adequacy across three specific sources: family, friends and significant others. Each item is scored on a 7-point rating scale ranging from *very strongly disagree* (1) *to very strongly agree* (7). By focusing on the subjective perceived support from three different sources, the MSPSS is a useful instrument in investigating how each source may be directly and differentially related to the mental health of depressed and non-depressed mothers. The scale has been found to be psychometrically sound in non-Western samples (Akhter et al., 2010; Eker and Arkar, 1995; Eker et al., 2000; Husain et al., 2006).

The subscale structure of the MSPSS distinguishes it from many other scales in that it considers three sources of support as separate sub groupings rather than only examining the objective or quantitative measurement of social support (Zimet et al., 1988). By focusing on subjective perceived support from different sources the MSPSS is a useful tool in investigating how support from each source may be directly and differently related to the reported severity of physical and psychological symptoms or how it may act as a buffer that moderates between stressful situations.

Eker et al. (Eker and Arkar, 1995; Eker et al., 2000) used translated version of MSPSS in Turkish population and have shown that it can be successfully used in non-western population showing finding similar to previous western studies (Zimet et al., 1988). We have used this instrument in our studies in Pakistan (Akhter et al., 2010; Husain et al., 2006; Rahman et al., 2008) with good psychometric properties.

#### 2.6.5 Statistical methods

**Fig. 1:**
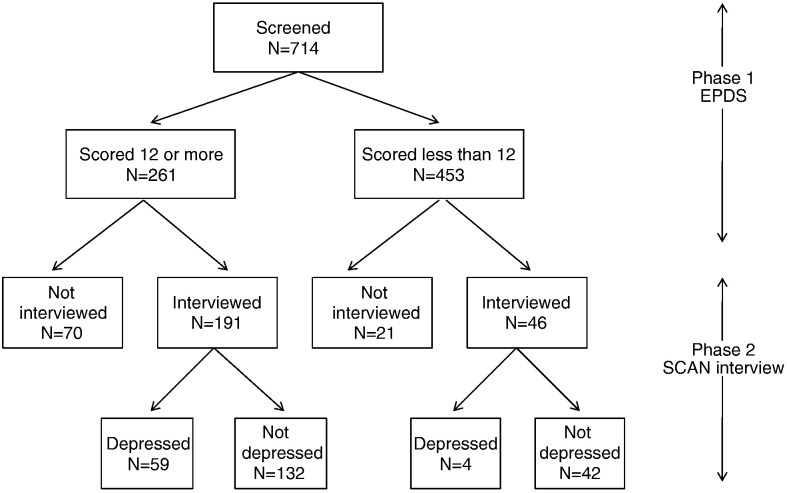
Flow of participants through two-phase antenatal screen and interviews.

A total of 714 women were screened in phase 1, of whom 261 scored 12 or more, and 191 of these took part in the phase-2 interviews, along with 46 of the 453 women who scored below 12 on the EPDS (Fig. 1). Thus in accordance with the methods for two-phase sampling studies, all analyses conducted on the phase-2 data used sampling weights, which were 261/191 for the high scorers and 453/46 for the low scorers. These were carried out using the survey sampling section within the statistical analysis software Stata (version 9, StataCorp LP, Texas, US).

The weighted prevalence of depression at antenatal assessment is presented. Depressed and non depressed mothers at the antenatal assessment were compared with respect to all the socio-demographic factors, marked difficulties and MSPSS subscales at baseline. For categorical variables, odds ratios with 95% confidence intervals and significance are presented, along with actual numbers of subjects in each category. For continuous variables, means, linearised standard errors, mean differences and significance are presented. Age and variables which were significant at p < 0.2 on the univariable analyses were then entered into a logistic regression analysis with depression at antenatal assessment as the dependent variable. The independent variables were the 3 MSPSS subscales, non health related marked difficulties, being 1st generation Pakistani, fluent in English, married, require permission to go out, marked social isolation, social support, dissatisfaction with the level of social support, and presence of an intimate confidant. A second analysis including the additional variable, whether the mother had previously suffered with nerves was conducted, and then a third, including instead the variable, whether the mother had previously been depressed. Odds ratios, 95% confidence intervals and significance values are presented, adjusted for the other variables in the same analysis.

Depressed and non depressed mothers at follow up interview were compared with respect to socio-demographic factors, marked difficulties and MSPSS subscales at baseline, using the same methods as for antenatal depression with appropriate sampling weights to account for the two-phase design, selection of subjects for the diagnostic interviews, and attendance at follow up interview. In addition, univariable analyses were carried out for reduction in difficulties scores between baseline and follow up for all difficulties, subject's own health, others' health, non health, environment and marital/other relationships.

Age and variables which were significant at p < 0.2 on the univariable analyses were then entered into a logistic regression analysis with depression at follow up as the dependent variable. Odds ratios, 95% confidence intervals and significance values from the logistic regression are presented. The independent variables were age, the 3 MSPSS subscales, non health related marked difficulties, others' health related marked difficulties, being fluent in English, marked social isolation, social support, and dissatisfaction with the level of social support. Second and third analyses including previous nerves and previous depression in turn were conducted as for the baseline analyses.

After the follow up interviews the women who were depressed at both antenatal and postnatal interviews (persistent depression) and those who were depressed at the first interview but not at the follow up interview (resolved depression) were identified. Univariable analyses were carried out to compare these two groups, with respect to socio-demographic factors, marked difficulties and MSPSS subscales at baseline, using Fisher's exact test for dichotomous variables, and the *t*-test for continuous variables. In addition to this, the reductions in difficulty scores were compared using the *t*-test. Only the MSPSS showed significant differences between the two groups, so logistic regression analyses similar to those above were not carried out. Effect sizes for the ‘key’ variables, reduction in non-health difficulties scores and, in particular, marital and other relationship difficulty scores were calculated.

## 3 Results

A total of 714 women were screened with the EPDS, of whom 261 (36.6%) scored 12 or more. Of these, 191 (73.2%) went into the second phase of the study. Reasons why 70 did not are as follows: the baby was delivered before an interview took place for 30 mothers, 2 babies died, 11 mothers refused, 11 were unavailable, 11 could not be contacted and 5 were not asked. A random selection of 67 (14.8%) of the low EPDS scorers were asked for the second phase interview, and 46 of these (69%) completed interviews. Of the 21 who did not, 3 babies were delivered before the interview, 2 mothers refused, 15 were unavailable, and 1 could not be contacted.

### 3.1 Prevalence of depression

Out of the 191 high EPDS scorers, 59 (30.9%) were found to be depressed on the SCAN interview, and 4 out of the 46 (8.7%) low scorers were depressed. There were no significant differences in mean EPDS scores between those who went into the second phase of the study and those who did not, within the high and low screen groups. All further analyses on data obtained at the second-phase interview were carried out using sampling weights (453/46 for low EPDS scorers and 261/191 for high EPDS scorers). The weighted prevalence of antenatal depression in this group of women was 16.8% with 95% confidence interval 14.1% to 19.6%.

### 3.2 Possible correlates of depression: univariable analyses

The depressed mothers were significantly more likely to be 2nd generation Pakistani, to require permission to go out, to be socially isolated, unsupported socially and to be dissatisfied with this. They were also significantly more likely to have marked difficulties, particularly non-health related ones (housing, financial and marital or other relationship difficulties), and were more likely to have previously suffered with “nerves”, depression or to have received psychological treatment, and they scored significantly lower on all 3 MSPSS subscales (Table 1). There was no significant difference between the depressed and non depressed mothers with respect to age, fluency in English, educational level, marital status, whether the pregnancy was planned, gravidity, number of existing children, whether the mother chats to others while out, had someone to talk to generally, or has a confidant.

The logistic regression analyses showed marked social isolation and marked non-health-related difficulties to be independent correlates of depression (Table 2). The odds ratio for marked social isolation was 6.37 (95% CI 2.46 to 16.5, p < 0.001), and only slightly changed to 6.27 and 6.32, respectively, when either previous nerves or depression were included in the independent variable list. The odds ratio for marked non health-related difficulties was 7.77 (95% CI 1.82 to 33.2, p = 0.006) and was also only very slightly changed with the addition of “nerves” or depression. Previous depression was not quite a significant predictor of depression at baseline interview (odds ratio 2.23, 95% CI 0.93 to 5.40), but previous “nerves” was significant (odds ratio = 2.58, 95% CI 1.04 to 6.39).

### 3.3 Depression at 6 months follow up

Follow up data was obtained on 208 (87.8%) of the 237 subjects with full baseline data, and of these, 47 (22.6%) were depressed at follow up. The 29 mothers who were not followed up gave the following reasons: 8 moved away, 3 withdrawn because of the baby's ill health, 1 withdrawn because the baby died, 2 refused, 9 lost contact, 1 withdrawn because of the mother's ill health, and 5 were unavailable. Ten (15.9%) of the 63 mothers who were depressed at baseline were lost to follow up, compared with 19 out of 174 (10.9%) of the non-depressed, Fisher's exact p = 0.37.

Univariable analyses for all the possible correlates of depression showed significant odds ratios with follow up depression at p < 0.05 for the following variables: marked difficulties at follow up, specifically non health (financial, marital, other relationship, and entrapment), not being married, social isolation, having previously suffered from “nerves” and depression, and all 3 MSPSS subscales. Additional variables which were entered into the subsequent multivariate logistic regression because they were significant at p < 0.2 were marked others' health related difficulties, marked difficulties involving humiliation, age 35 or over, fluency in English, lack of social support, and dissatisfaction with this (univariable results not tabulated, but available on request).

Multivariate logistic regression with follow up depression as the dependent variable showed that marked social isolation and marked non health related difficulties at antenatal assessment were significant independent predictors of depression at follow up (Table 2, right hand 3 columns). The odds ratio for marked social isolation was 9.00 (95% CI 2.44 to 33.2, p = 0.001), and the odds ratio for marked non health-related difficulties was 23.7 (95% CI 4.47 to 125, p = 0.001). The inclusion of previous “nerves” did not alter these results, but having previously suffered from “nerves” was also a significant predictor of follow up depression (odds ratio 3.12, 95% CI 1.23 to 7.90). The inclusion of previous depression did not alter the results, and previous depression was not a significant predictor of depression at follow up (odds ratio 2.17, 95% CI 0.84 to 5.55).

### 3.4 Persistent depression

Of the 208 mothers who had a diagnostic interview at both baseline and follow up, 134 were not depressed at either interview, 21 were not depressed at baseline but became depressed at follow up, 27 were depressed at baseline but not at follow up, and 26 were depressed at both interviews. Univariable analyses of all the possible correlates of depression previously considered, comparing the 26 mothers with persistent depression with the group of 27 whose depression resolved are shown in Table 3. Only the MSPSS subscales for family and significant other showed significant differences between the two groups at p < 0.05. Therefore logistic regression analyses similar to those for depression at baseline were not carried out.

The lack of significant effects could have been partly explained by the small sample size for these comparisons, but the odds ratio for marked non-health difficulties is very close to 1, suggesting that the difference is not likely to be clinically significant either. The proportion of subjects with these difficulties is very similar in the persistent and resolved groups (31% vs. 30%, Table 3). The reduction in difficulties scores did not even approach statistical significance, for either the total score or for any of the subdivisions of difficulty types. The effect size for reduction in non health difficulties scores is 0.40 and for marital and other relationships is 0.37, both of which are regarded as small to medium. Sample sizes of 116 subjects in each of the persistent and resolved groups would be required to have 80% power to detect the latter as being statistically significant on a two-sided 5% *t*-test.

## 4 Discussion

Using EPDS the prevalence of antenatal depression was 30.9%. The weighted prevalence in this group was 16.8%. Depressed mothers were significantly more likely to have marked difficulties particularly non health related such as housing, financial and marital. Furthermore they were socially isolated, had poor social support and were dissatisfied with the support they received. In the group where depression was persistent after 6 months the MSPSS subscales for family and significant other and the total scores showed significant differences between the groups at p < 0.05.

### 4.1 Prevalence of depression

The prevalence of antenatal depression within this sample was 16.9% (weighted; non-weighted was 30.9%) which is much lower than the 25% prevalence of antenatal depression reported from Pakistan (Rahman et al., 2003; Rahman et al., 2004). A recent systematic review has reported 6.5% to 12.9% combined point estimate prevalence of depression during 3rd trimester of pregnancy (Gavin et al., 2005). Faisal-Cury et al. (2004) reported 19.6% Brazilian women to be depressed in antenatal period.

### 4.2 Risk factors for depression during the perinatal period

Faisal-Cury et al. (2004) found antenatal depression to be associated with low income, greater number of previous abortions and being single. In our study depressed women had experienced more non health related difficulties (housing, financial and marital), and were more likely to have previously suffered with depression or to have received psychological treatment. In our study there was no significant difference between depressed and non depressed mothers with respect to age, fluency in English, educational level, marital status or number of existing children.

Chandran et al. (2002) found 16.2% women in India to be depressed in the antenatal period and 19.8% in the post natal period. The authors identified the following risk factors for the incidence of depression: low income, adverse life events in the previous year, problem with in-laws, poor relationship with parents, birth of a daughter and lack of physical help. Our results did not show problems with in-laws, poor relationship with parents, giving birth to a daughter or lack of physical help to be associated with postnatal depression. Our findings in Manchester are comparable to our results in Pakistan (Husain et al., 2006) where we did not find previously reported risk factors like female gender of the infant and low level of education to be associated with postnatal depression however, social stresses showed strong association.

Our findings regarding the risk factors are somewhat similar to Cooper et al. (1999) results in South Africa. They reported no differences with maternal age, marital status, infant gender, parity or mode of delivery. They did not find postpartum depression to be significantly related to the availability of practical support. However, regular emotional and practical support from partner was reported by 58% non-depressed mothers and only 34% depressed mothers p < 0.01.

Rahman et al. (2003) in their study in Pakistan reported that depressed mothers were more likely to experience threatening life events. The main events in their study were; main earning member of the family being made unemployed. The difficulties were in the area of finance, housing, relationship, difficulties with a significant member of extended family and severe marital problems. The authors did not find any association with bereavement, major illness in the family or lack of confiding relationship.

Zelkowitz et al. (2004) found high rates of postnatal depression in immigrant mothers in Canada. In their study length of stay in the host country was associated with high EPDS scores. Those who had lived in Canada less than 5 years had higher scores. In our results the depressed mothers were more likely to be 2nd generation Pakistani. None of the participants had any marked immigration difficulties at base line.

### 4.3 Persistent depression

Our study looked at the role of established risk factors for postnatal depression. Patel et al. (2003) found risk of persistent postnatal depression in the mothers who had experienced marital violence and the risk was far greater if the infant was a female. Risks were lower if the new born was a boy; this is in contrast to our current findings. Our results are similar to Rahman et al. (2003) who reported that depressed women with poor social support were depressed during antenatal period and remained depressed after birth, rather than new onset of depression. Gotlib et al. (1989) found persistent depression in pregnancy and postpartum period to be associated with younger age, lower level of education and greater number of children in their house hold. Seguin et al. (1995) reported persistent depression in pregnant women to be strongly associated with chronic stressors and difficult life conditions such as inadequate housing and financial problems. Stressors in our sample were more specific to Pakistani women living in the UK with marked social isolation in addition to other more general non health related difficulties like marital difficulties, housing and financial problems.

### 4.4 Social support

O'Hara and Swain (1996) found that depressed mothers reported less emotional and practical support from the spouses than non depressed mothers. The depressed mothers reported less marital satisfaction. These findings are similar to our results. Depressed mothers in our sample scored low on all 3 MSPSS subscales.

Similarly Rahman et al. (2003) and Mumford et al. (2005) in their studies in Pakistan reported depression to be negatively associated with social support from the extended family. We have also found the risk of depression to be less when living in an extended family it is possible that if there is a need of extended families to be around it can be a protective factor.

The results of our study are similar to the reports from Canada (Zelkowitz et al., 2004) where high EPDS scores in immigrant women born outside of Canada were associated with experience of more stressful life events and less social support. The depressed women reported poor relationship with the partner.

Strengths of the study include the sampling method that limited the risk of recruitment bias, and the blinding of the diagnostic interviewers to the EPDS score. While previous researchers have questioned the validity of diagnosis of depression during pregnancy (Matthey and Ross-Hamid, 2011), we used a two phase design whereby the diagnosis was confirmed by a trained clinician using a standard diagnostic interview schedule. We used previously validated instruments for screening and robust instruments to measure depression and details of life events and difficulties. The interviews were conducted by female bilingual researchers coming from the same ethnic background. The sample was collected from two antenatal clinics and all women with an identifiable Pakistani name were approached. This is the only longitudinal study to our knowledge looking at the prevalence and risk factors of perinatal depression in Pakistani women living in UK. The advantage of this sample is that the sample is unaffected by treatment seeking. We had a high response with a very good follow up rate (87.5%).

Limitations of the study were the lack of formal reliability testing between the diagnostic interviewers but this was not a major concern as all researchers were trained at a WHO approved training centre. The life events and difficulties were recorded in majority of cases, after the onset of depression, we were able to study the social problems before the onset of depression only in a small number of cases and hence the study offers limited information about the aetiology of depression in this group. Because of the prospective design we were able to assess the factors associated with persistent depression. However, the numbers were small and it was not possible to draw definite conclusions about the factors associated with persistence of depression or the factors associated with the onset of new cases at follow-up. We have to be cautious when calling this sample a representative sample. There could be a possible bias as the research assistant attended the antenatal clinics twice a week and therefore women coming on other days were missed. Therefore the sample being truly representative of the general population may be debatable. Use of 2 stage screening method can be a potential limitation as only small number of low scorers was interviewed for the 2nd phase of the study. Ideally all mothers should have been interviewed but we did not have the resources to do so.

Although SCAN has been translated and used in Pakistani population in Pakistan and people of Pakistani origin in the UK however it is based on western psychiatry. Expression of distress and its consequences may be different in different cultures. It has been debated whether or not instruments designed in one system are applicable to other cultures. SCAN and LEDS were administered by the same interviewer. There is a possibility of interviewer bias in rating and reporting of life events and difficulties as the interviewer was aware of the subject's SCAN diagnosis. This could lead to increased number of severe difficulties in the depressed group. This issue is addressed as the rating of events and difficulties is decided by an experienced group of LEDS raters blind to the participant's diagnosis.

### 4.5 Further research

There is a constant rise in proportion of births to mothers born outside the UK: 23% in 2007 compared to 13% in 1997 (National Census Figures, 2009). The birth rate for native English women is 1.1 children while the birth rate for Pakistani women is more than triple (National Census Figures, 2009) There are major inequalities in maternal health and now there is a call for tailored maternity services to improve access to care for women from ethnic minorities (Knight et al., 2009). The findings of this study highlight the importance of social factors particularly social isolation and its association with depression in pregnant British Pakistani women. Further prospective studies also looking at biological and genetic factors would be needed to fully understand the factors associated with onset and persistence of perinatal depression in this population. Furthermore, studies looking at prevention and treatment of depression in this group are warranted.

## Role of funding source

Funding for this study was provided by Medical Research Council (MRC); the MRC had no further role in study design; in the collection, analysis and interpretation of data; in the writing of the report; and in the decision to submit the paper for publication.

## Conflict of interest

I can affirm that all the authors do not have any conflict of interest whether it is actual or potential conflict of interest including any financial, personal or other relationships with other people or organisations within three (3) years of beginning the work submitted that could inappropriately influence, or be perceived to influence, their work.

## Figures and Tables

**Table 1:** Possible correlates of depression at baseline.

Possible correlates of depression	Depressed (n = 63) % (n)	Not depressed (n = 174) % (n)	Odds ratio	95% CI	Sig
1st generation Pakistani	62.9% (49)	82.6% (142)	0.35	0.13 to 1.00	0.049
Fluent in English	77.9% (42)	61.9% (103)	2.16	0.96 to 4.87	0.062
Married	96.5% (60)	99.0% (170)	0.29	0.06 to 1.39	0.12
Require permission to go out	17.1% (15)	5.4% (11)	3.62	1.16 to 11.3	0.027
Marked isolation	51.7% (33)	24.6% (51)	3.28	1.36 to 7.94	0.009
Receive social support	72.4% (45)	89.5% (147)	0.31	0.11 to 0.85	0.024
Dissatisfied with social support	29.8% (20)	13.8% (35)	2.66	1.02 to 6.96	0.045
Have an intimate confidant	92.0% (56)	97.4% (169)	0.31	0.06 to 1.48	0.14
Previously suffered from nerves	54.0% (35)	19.1% (52)	4.97	2.05 to 12.0	< 0.001
Previously suffered from depression	48.3% (30)	17.0% (43)	4.55	1.85 to 11.2	0.001
Previously received psychological treatment	25.3% (16)	5.6% (18)	5.74	1.90 to 17.3	0.002
Marked non-health difficulty	29.8% (20)	8.4% (18)	4.62	1.58 to 13.5	0.005
Any marked difficulty	34.4% (24)	10.8% (22)	4.34	1.60 to 11.8	0.004

Continuous variables	Mean (se)	Mean (se)	diff	95% CI	Sig

Age^a^	29.0 (1.04)	28.9 (0.65)	0.2	− 1.9 to 2.3	0.85
MSPSS subscales^b^:					
Total	55.3 (3.84)	69.0 (1.27)	− 13.6	− 21.5 to − 5.8	0.001
Family	4.71 (0.45)	5.92 (0.12)	− 1.2	− 2.1 to − 0.3	0.010
Friends	3.98 (0.39)	5.24 (0.18)	− 1.2	− 2.1 to − 0.4	0.003
Significant other	5.14 (0.38)	6.09 (0.10)	− 1.0	− 1.7 to − 0.2	0.013

Within group percentages, odds ratios, confidence intervals and significance values are weighted according to inverse sampling weights. Actual numbers of subjects are presented. 95% CI = 95% confidence interval; Diff = mean difference; Se = linearised standard error.

a Age is unknown for 1 depressed subject.

b MSPSS data missing for 10 depressed and 27 non-depressed subjects.

**Table 2:** Logistic regression analyses with depression at baseline and at follow up as the dependent variables.

Possible risk factor	Dependent variable depression at baseline			Dependent variable depression at follow up		
	Odds ratio	95% CI	Sig.	Odds ratio	95% CI	Sig.
Age	0.99	0.91 to 1.08	0.85	1.03	0.93 to 1.14	0.55
Marked social isolation	6.37	2.46 to 16.5	< 0.001	9.00	2.44 to 33.2	0.001
Others' health related marked difficulties^a^	–	–	–	11.9	1.08 to 131	0.043
Non health related marked difficulties	7.77	1.82 to 33.2	0.006	23.7	4.47 to 125	< 0.001
MSPSS family	0.71	0.44 to 1.14	0.15	0.75	0.46 to 1.21	0.24
MSPSS friends	0.76	0.52 to 1.11	0.15	0.68	0.44 to 1.05	0.081
MSPSS significant other	0.97	0.65 to 1.43	0.86	0.82	0.54 to 1.24	0.35

a Variable not included as p > 0.2 on univariate analyses. The variables 1st generation Pakistani, fluency in English, married, require permission to go out, social support, dissatisfaction with level of social support, and having an intimate confidant were not significant in either logistic regression analysis.

**Table 3:** Possible risk and protective factors for persistent depression among mothers who were depressed at antenatal assessment.

Protective and risk factors	Persistent depression (n = 26) % (n)	Resolved depression (n = 27) % (n)	Odds ratio	95% CI	Sig
1st generation Pakistani	80.8% (21)	74.1% (20)	1.47	0.40 to 5.40	0.75
Speak English	69.2% (18)	70.4% (19)	0.95	0.29 to 3.06	1.0
Fluent in English 1	69.2% (18)	73.1% (19)	0.83	0.25 to 2.76	1.0
Married 1	92.3% (24)	96.2% (25)	0.48	0.04 to 5.65	1.0
Require permission to go out	23.1% (6)	22.2% (6)	1.05	0.29 to 3.80	1.0
Marked isolation	53.8% (14)	51.9% (14)	1.08	0.37 to 3.19	1.0
Receive social support	76.9% (20)	70.4% (19)	1.40	0.41 to 4.81	0.76
Dissatisfied with social support	26.9% (7)	37.0% (10)	0.63	0.19 to 2.01	0.56
Have an intimate confidant	92.3% (24)	88.9% (24)	1.50	0.23 to 9.80	1.0
Previously suffered from nerves	69.2% (18)	55.6% (15)	1.80	0.58 to 5.56	0.40
Previously suffered from depression	53.8% (14)	51.9% (14)	1.08	0.37 to 3.19	1.0
Previously received psychological treatment	30.8% (8)	29.6% (8)	1.06	0.33 to 3.41	1.0
Marked non-health difficulty	30.8% (8)	29.6% (8)	1.06	0.33 to 3.41	1.0
Any marked difficulty	42.3% (11)	33.3% (9)	1.47	0.48 to 4.48	0.58
Age 2	29.0 (5.93)	28.5 (4.65)	0.5	− 2.5 to 3.4	0.74
MSPSS Family 3	3.9 (1.7)	5.3 (1.0)	− 1.5	− 2.3 to − 0.6	0.002
MSPSS Friends	4.3 (1.9)	4.3 (2.0)	0.1	− 1.1 to 1.2	0.93
MSPSS Significant other	4.8 (1.6)	5.7 (1.3)	− 0.9	− 1.8 to − 0.03	0.044
Reduction in difficulties scores:					
Total	0.08 (3.28)	1.04 (2.82)	− 1.0	− 2.6 to 0.7	0.26
Subject's own health	0.04 (0.34)	0.04 (0.34)	0.001	− 0.2 to 0.2	0.99
Others' health 4	0.15 (1.19)	0.00 (0.00)	0.2	− 0.3 to 0.6	0.52
Non health	− 0.12 (2.92)	1.00 (2.72)	− 1.1	− 2.7 to 0.4	0.16
Environment	0.12 (1.51)	0.37 (1.50)	− 0.3	− 1.1 to 0.6	0.54
Marital and other relationships	− 0.23 (2.63)	0.63 (2.08)	− 0.9	− 2.2 to 0.4	0.19

95% CI = 95% Confidence Interval; Diff = mean difference.

1 Missing data for 1 mother in the resolved group.

2 Age is unknown for 1 mother in the persistent group.

3 MSPSS data is available for 23 mothers in the persistent group and 21 in the resolved group.

4 Levene’s test was significant, and the unequal variance version of the t-test was used.
